# Supplementary material for: Protein Disulfide Isomerase and Extracellular Adherence Protein Cooperatively Potentiate Staphylococcal Invasion into Endothelial Cells
Source: Microbiol Spectr. 2023 Mar 30;11(3):e03886-22. doi: 10.1128/spectrum.03886-22 (PMC10269700; doi:10.1128/spectrum.03886-22)
Supplement: Supplemental file 1 — Supplemental material. Download spectrum.03886-22-s0001.pdf, PDF file, 0.6 MB [file spectrum.03886-22-s0001.pdf]

Supplemental figures

Protein disulfide isomerase and extracellular adherence protein (Eap) cooperatively potentiate staphylococcal invasion into endothelial cells

Short title: Eap promotes bacterial invasion by stimulation of PDI

Marleen Leidecker[¶], Anne Bertling[¶], Muzaffar Hussain, Markus Bischoff, Johannes A. Eble, Anke C. Fender, Kerstin Jurk, Christine Rumpf, Mathias Herrmann, Beate E. Kehrel[&], Silke Niemann[&]

Corresponding authors:
E-mail: silke.niemann@uni-muenster.de, kehrel@uni-muenster.de

[¶]These authors contributed equally to this work. The order of the authors was determined by the chronological order of the work on the study; Marleen Leidecker took over the work from Anne Bertling.
[&]These senior authors also contributed equally to this work.

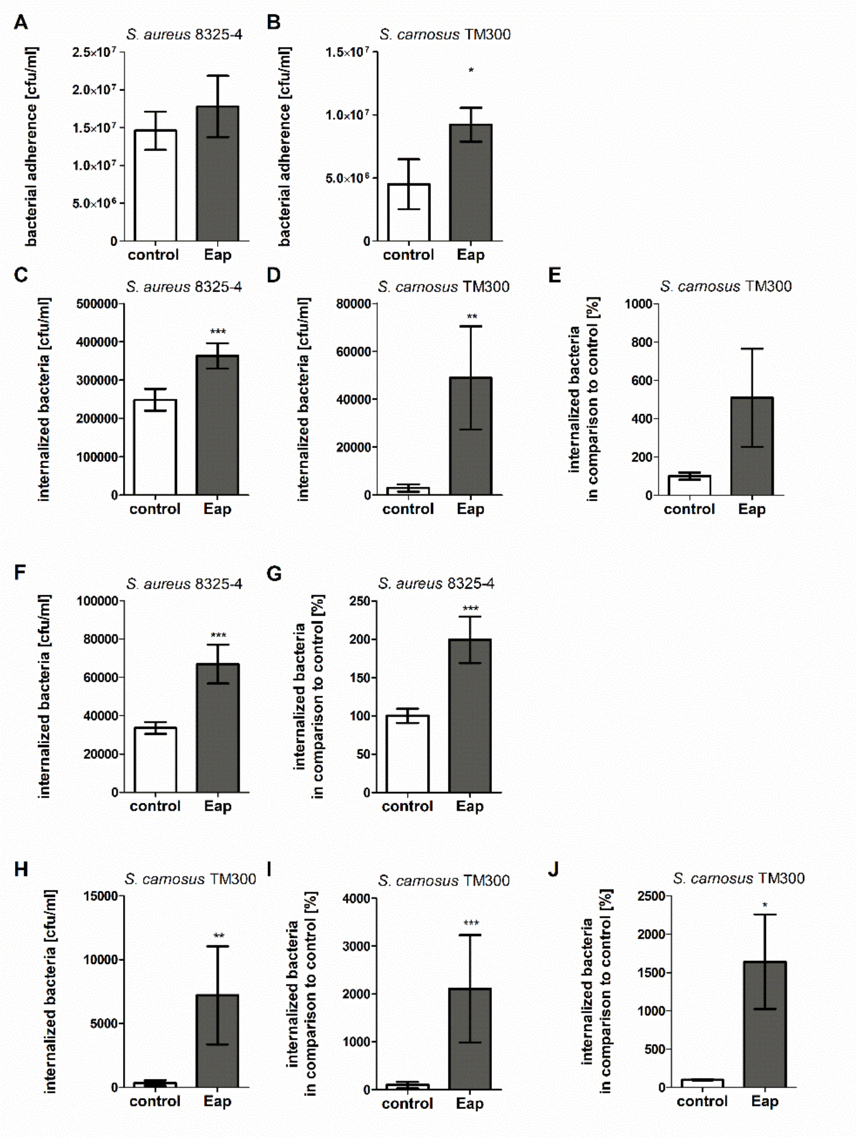

Supplemental Fig. S1: Eap enhances staphylococcal adherence to and internalization in endothelial cells.

(A) Adhesion of *S. aureus* 8325-4 and **(B)** *S. carnosus* TM300 to HMEC-1 in absence and presence of 20 µg/ml recombinant Eap. Cells were incubated with an MOI of 50. One hour post infection unbound bacteria were washed away with PBS. Host cells were lysed with ice-cold distilled water in order to release extra- and intracellular bacteria and number of bacteria was assessed by plate counting. Data represent the means ± SD from absolute numbers of four (*S. aureus*) or three (*S. carnosus*) independent experiments. *p<0.05, unpaired t test. **(C)** Internalization of *S. aureus* 8325-4 and **(D)** *S. carnosus* TM300 in HMEC-1 in absence and presence of 20 µg/ml recombinant Eap. Cells were incubated with an MOI of 50. One hour post infection, extracellular staphylococci were removed by lysostaphin treatment. Host cells were lysed with ice-cold distilled water in order to release intracellular bacteria and number of bacteria was assessed by plate counting. Data represent the means ± SD from absolute numbers from five independent experiments. ***p≤0.001, **p≤0.01, unpaired t test. **(E)** Internalization of *S. carnosus* TM300 in HMEC-1 in absence and presence of 20 µg/ml native Eap. Numbers of bacteria in control cells were set to 100%. Data represent the means ± SD from absolute numbers from three independent experiments, unpaired t test. **(F)** Internalization of *S. aureus* strain 8325-4 (absolute numbers) and **(G)** (numbers of bacteria in control cells were set to 100%) in EA.hy926, recombinant Eap. **(H)** Internalization of *S. carnosus* TM300 (absolute numbers) and **(I)** (numbers of bacteria in control cells were set to 100%) in EA.hy926 cells, recombinant Eap. **(J)** Internalization of *S. carnosus* TM300 in EA.hy926 in absence and presence of 20 µg/ml native Eap. Numbers of bacteria in control cells were set to 100%. Data represent the means ± SD from at least three independent experiments. ***p≤0.001, **p≤0.01, *p<0.05 unpaired t test.

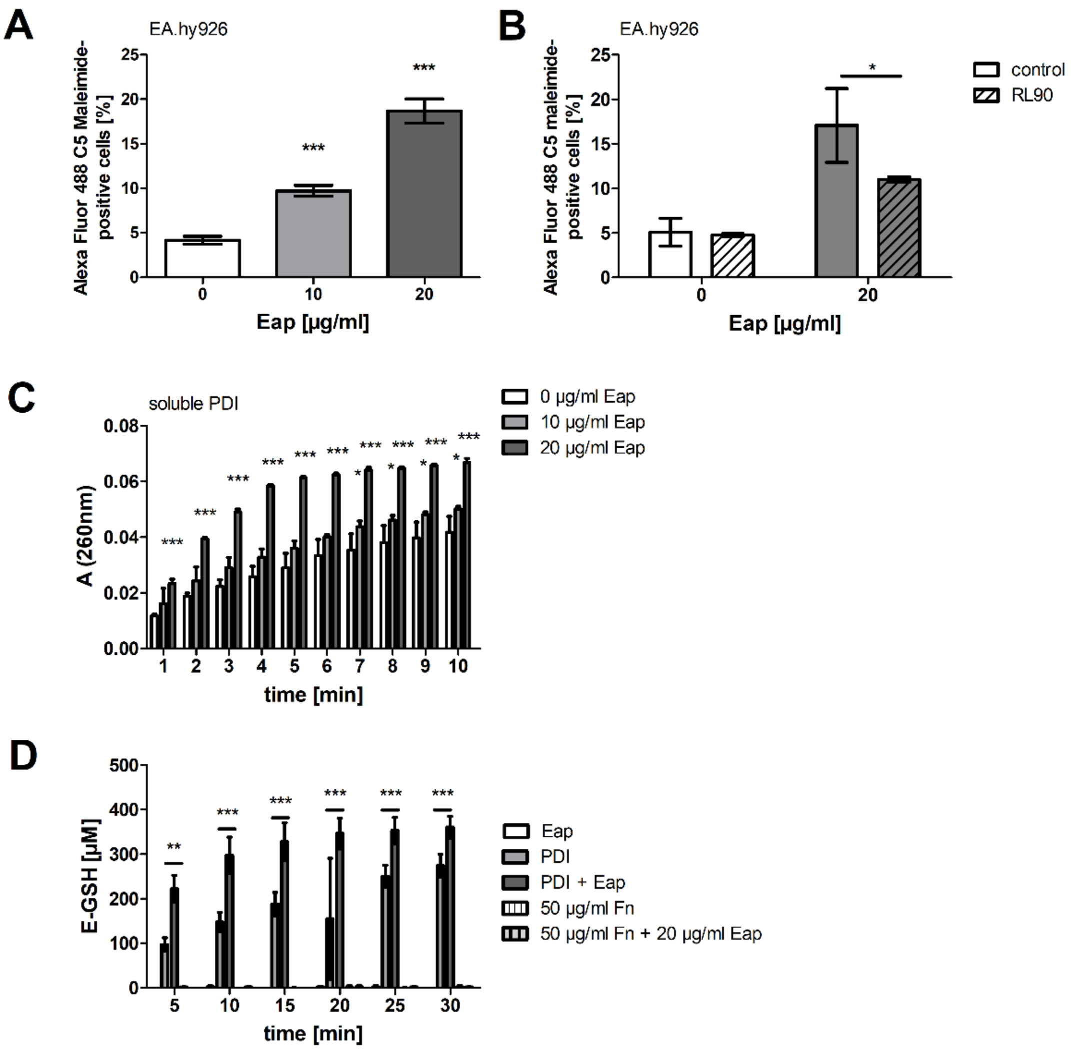

Supplemental Fig. S2: Eap promotes protein disulfide activity on the surface of eukaryotic cells.

(A) Eap-stimulated abundance of free ecto-sulfhydryls on the surface of EA.hy926 cells was detected by the binding of the thiol-reactive dye Alexa Flour 488 C_5 maleimide and measured by flow cytometry. Native Eap, data represent the mean ± SD of three independent experiments. ***p≤0.001; one-way ANOVA followed by Dunnett's multiple-comparison test. **(B)** Inhibition of Eap-promoted abundance of ecto-sulfhydryls on EA.hy926 by anti-PDI antibody RL90 (10µg/ml) detected with Alexa Flour 488 C_5 maleimide. Recombinant Eap, data represent the mean ± SD of three independent experiments. *p<0.05, two-way ANOVA followed by Bonferroni posttest. **(C)** Influence of Eap on kinetics of PDI-catalyzed oxidative refolding of scrambled RNAse A, determined as ΔA_{260nm}. Eap was preincubated with soluble PDI before PDI was incubated with scrambled RNAse A. Recombinant Eap, data represent the mean ± SD of three independent experiments. ***p≤0.001, *p<0.05; two-way ANOVA followed by Bonferroni posttest, comparison to 0 µg/ml Eap. **(D)** Influence of Eap (20 µg/ml) on kinetics of Di-E-GSSG reduction to E-GSH catalyzed by soluble PDI (200 nM) or by fibronectin (50 µg/ml). Native Eap, data represent the mean ± SD of three independent experiments. ***p≤0.001, **p≤0.01; two-way ANOVA followed by Bonferroni posttest; for better readability, only significant differences between PDI and PDI + Eap are indicated.

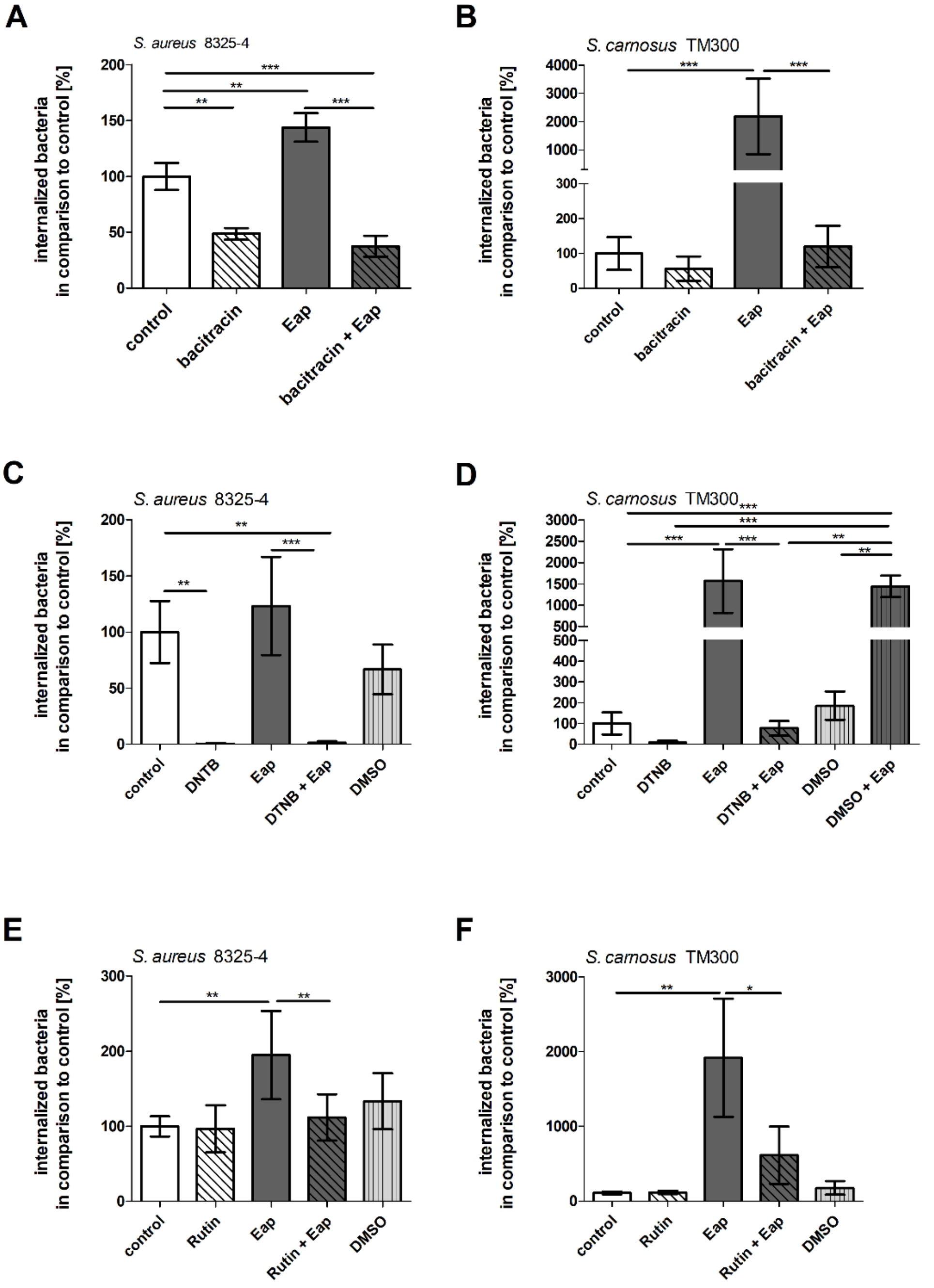

Supplemental Fig. S3: Inhibition of protein disulfide isomerase reverses the enhancing effect of Eap on staphylococcal internalization.
Internalization of *S. aureus* 8325-4 and *S. carnosus* TM300 as indicated in HMEC-1 cells in absence and presence of 20 µg/ml recombinant Eap. Cells were pre-incubated with **(A/ B)** bacitracin (10mM), **(C/ D)** dithiobis-nitrobenzoic acid (DTNB, 10 mM; DMSO, 1% v/v as vehicle control), **(E/ F)** Rutin (60 nM; 0.6% v/v as vehicle control) 30 minutes before stimulation with Eap. In case of bacitracin, host cells were washed before addition of bacteria to remove bacitracin. Cells were incubated with an MOI of 50. One hour post infection, extracellular staphylococci were removed by lysostaphin treatment. Number of intracellular bacteria was assessed by plate counting. Numbers of bacteria in control cells were set to 100%. Recombinant Eap, data represent the mean ± SD of at least three independent experiments. ***p≤0.001, **p≤0.01, *p<0.05; one-way ANOVA followed by Bonferroni posttest.

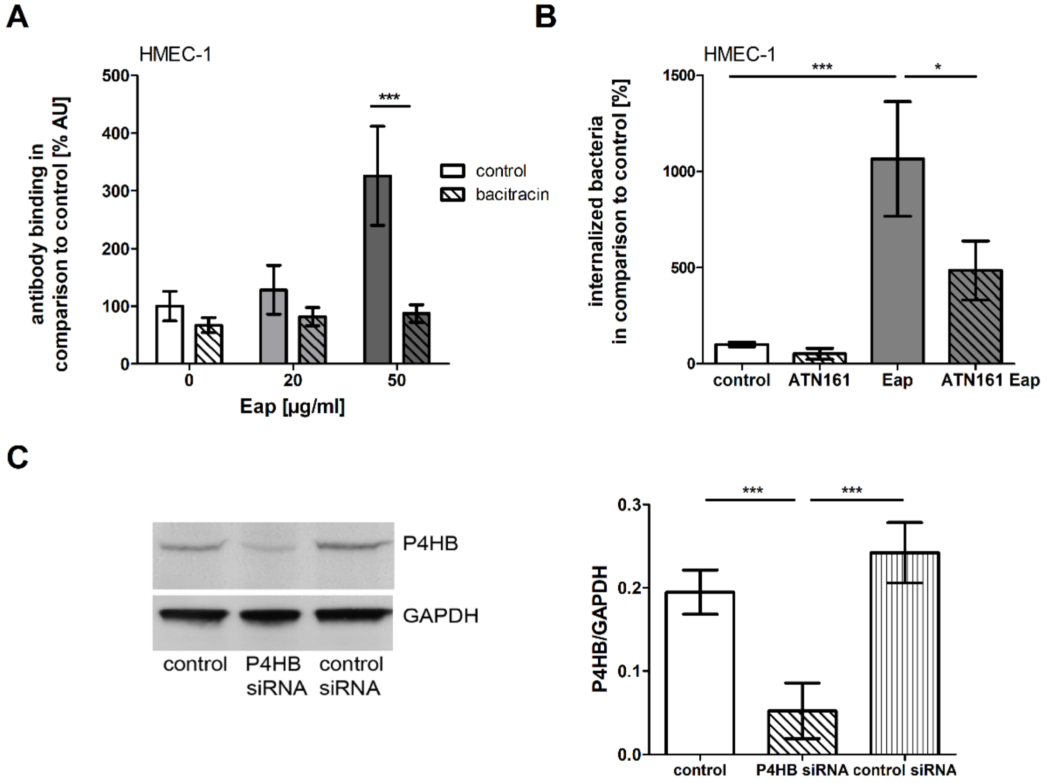

Supplemental Fig. S4: Eap enhances activation of β1 integrin to cells in a thiol isomerase-dependent manner, affecting staphylococcal uptake

(A) Binding of FITC-labelled antibody against activated anti-integrin β1 to detached HMEC-1 cells was determined by flow cytometry after incubation of cells with native Eap native Eap and/ or or bacitracin (10mM) added to the cells 15 min before addition of Eap, binding of antibody to control (0 µg/ml Eap) was set to 100%. Data represent the means ± SD of at least three independent experiments, ***p≤0.001, two-way ANOVA followed by Bonferroni posttest. **(B)** Internalization of *S. carnosus* TM300 HMEC-1 cells in absence and presence of 20 µg/ml Eap. Cells were pre-incubated 10 µM ATN161 30 minutes before stimulation with Eap. Cells were incubated with an MOI of 50. One hour post infection, extracellular staphylococci were removed by lysostaphin treatment. Number of intracellular bacteria was assessed by plate counting. Numbers of bacteria in control cells were set to 100%. Recombinant Eap, data represent the mean ± SD of three independent experiments. ***p≤0.001, *p<0.05; one-way ANOVA followed by Bonferroni posttest. **(C)** siRNA P4HB knock down (10 pM siRNA) in HMEC-1 cells was confirmed by Western Blotting. Data are means ± SD from Western blot quantification of three independent experiments. ***p≤0.001; one-way ANOVA followed by Bonferroni posttest.
